# Speaker Sex Perception from Spontaneous and Volitional Nonverbal Vocalizations

**DOI:** 10.1007/s10919-018-0289-0

**Published:** 2018-10-20

**Authors:** Nadine Lavan, Abigail Domone, Betty Fisher, Noa Kenigzstein, Sophie Kerttu Scott, Carolyn McGettigan

**Affiliations:** 1 Department of Psychology, Royal Holloway, University of London, Egham Hill, Egham, TW20 0EX UK; 2 Department of Speech, Hearing and Phonetic Sciences, University College London, London, UK; 3 Institute of Cognitive Neuroscience, University College London, London, UK

**Keywords:** Speaker sex, Nonverbal vocalizations, Laughter, Fundamental frequency, Crying, Spontaneous

## Abstract

In two experiments, we explore how speaker sex recognition is affected by vocal flexibility, introduced by volitional and spontaneous vocalizations. In Experiment 1, participants judged speaker sex from two spontaneous vocalizations, laughter and crying, and volitionally produced vowels. Striking effects of speaker sex emerged: For male vocalizations, listeners’ performance was significantly impaired for spontaneous vocalizations (laughter and crying) compared to a volitional baseline (repeated vowels), a pattern that was also reflected in longer reaction times for spontaneous vocalizations. Further, performance was less accurate for laughter than crying. For female vocalizations, a different pattern emerged. In Experiment 2, we largely replicated the findings of Experiment 1 using spontaneous laughter, volitional laughter and (volitional) vowels: here, performance for male vocalizations was impaired for spontaneous laughter compared to both volitional laughter and vowels, providing further evidence that differences in volitional control over vocal production may modulate our ability to accurately perceive speaker sex from vocal signals. For both experiments, acoustic analyses showed relationships between stimulus fundamental frequency (F0) and the participants’ responses. The higher the F0 of a vocal signal, the more likely listeners were to perceive a vocalization as being produced by a female speaker, an effect that was more pronounced for vocalizations produced by males. We discuss the results in terms of the availability of salient acoustic cues across different vocalizations.

## Introduction

Listeners can determine a speaker’s sex from their vocal signals with high accuracy (Coleman 1971; Lass et al. 1976). Speaker sex can be assessed rapidly, with listeners being able to identify sex from vowel segments lasting under 2 glottal cycles (i.e., two cycles of the vocal folds in the larynx opening and closing to produce a buzzing sound; Owren et al. 2007). Listeners can furthermore successfully perceive speaker sex from drastically degraded or manipulated vocal signals, such as sine-wave^1^ speech and noise-vocoded speech^2^ with as few as 3 channels (Gonzalez and Oliver 2005).

The perceptual cues assumed to allow listeners to distinguish male from female voices are linked to sex-specific anatomical features of the vocal tract: Due to the pronounced sexual dimorphism of the human larynx and vocal folds, males on average tend to have longer and thicker vocal folds than females, as well as longer vocal tracts (Titze 1989). These two features mainly lower the fundamental frequency of the voice (F0, broadly perceived as voice pitch) and affect the spacing of the formants in vocal signals. Thus, males and females differ anatomically which affects the source signal (i.e., F0; buzzing sound created through the vibration of the vocal folds in the larynx) and the filter characteristics (i.e., formants; resonant characteristics of the vocal tract, determined by its shape and size; see source-filter-model; Fant 1960), making male and female voices relatively distinct from each other. Studies using perceptual judgements and computational approaches have indeed shown that acoustic cues, such as these differences in F0 and formant characteristics, are crucial for determining speaker sex from vocal signals that have been produced in a neutral voice (Bachorowski and Owren 1999; Skuk and Schweinberger 2014). The salience of these cues for speaker sex identification is highlighted in a study by Mullennix et al. (1995): the authors shifted F0 and formant frequencies in vocalizations and were thereby able to successfully create continua of vocalizations that were perceived by listeners to morph from male to female.

While both formant frequencies and F0—alongside other acoustic measures—play an important role in determining speaker sex, it has been argued that F0 may be the more salient cue for speaker sex judgements: Lass et al. (1976) have shown that removing the source signal (which encodes F0 information) by using whispered speech affects participants’ judgements of speaker sex more drastically than when stimuli are low-pass filtered (thus removing all filter information, and therefore all formants [apart from F0]). Several other studies comparing the contributions of formant frequencies and F0 to speaker sex perception also conclude that F0 is the more salient acoustic cue (Gelfer and Bennett 2013; Poon and Ng 2015; Whiteside 1998). Honorof and Whalen (2010) reported that when F0 is volitionally manipulated by a speaker within their natural range when producing isolated vowels, miscategorizations of speaker sex occur at the extremes of the F0 range, with high F0 being identified as female and low F0 as male. Similarly, Bishop and Keating (2012) report that in the context of variable pitch, male voices are most accurately identified when the vocal signals produced have an F0 that is lower than 200 Hz while the reverse is true for female voices. These studies show that changes in salient acoustic cues, through explicit volitional voice modulations, as well as synthetic manipulations of the stimuli, can affect the accuracy of speaker sex judgements from voices.

Thus, sex perception from voices can be affected using stimuli designed to be ambiguous, be they artificially manipulated signals or volitionally produced phsyiological extremes. However, voices and their acoustic properties—such as the F0—are highly variable and flexible in their everyday use (Lavan et al. 2018c): speakers dynamically modulate their voices depending on the speaking environment, communicative intent or physiological and psychological state. One major modulator of the voice is the person’s affective state: a large body of literature has shown that affective tone in vocal signals impacts the acoustic and perceptual properties of these signals, compared to neutral vocalizations—or indeed between different emotional vocalizations (see Juslin and Laukka 2003; Sauter et al. 2010). For example, modulations of F0, speech rate (for emotional speech), spectral features, periodicity and amplitude (see acoustic analyses in the “Appendix” section for descriptions of these measures) have all been reported in comparisons of emotional and neutral vocalizations. Additionally, some research has recently shown that spontaneous (emotional) vocalizations differ from volitionally produced exemplars of the same type of vocalization, most prominently for laughter: based on differences in their production mechanisms, significant differences in acoustic properties (including F0) and affective properties have been reported for volitional and spontaneous vocalizations (Bryant and Aktipis 2014; Lavan et al. 2016a; Ruch and Ekman 2001). Notably, two recent studies have also reported reduced performance for a speaker discrimination task for spontaneous laughter (contrasted with volitional laughter; Lavan et al. 2016b, 2018a).

The above research on emotional vocalizations, and the natural variability within them, reflects a general movement within current theoretical and empirical approaches to the study of nonverbal behavior. It is now more broadly acknowledged that “one size fits all” labels such as “laughter” and “crying” are often insufficient to account for the complex context-dependency of natural behaviors, and how they are perceived (Anikin and Lima 2017; Martin et al. 2017; Sauter 2010; Sauter and Scott 2007). Importantly, evidence suggests that variations within nonverbal behaviors not only have an impact on (affective) state evaluations, but also on the perception of stable indexical characteristics (i.e., identity); therefore, to develop better models of how humans perceive other people, we must understand how this takes place across the range of natural human behaviors.

**Fig. 1:**
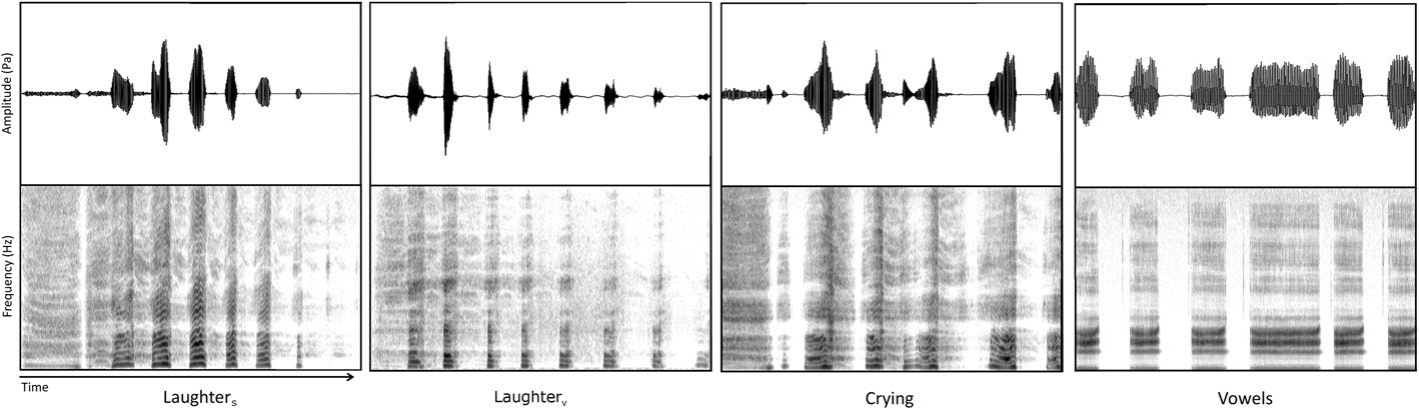
Waveforms (top panels) and spectrograms (bottom panels) of the vocalization types used in Experiment 1 and 2: Spontaneous Laughter (Laughter_S_), Volitional Laughter (Laughter_V_), Spontaneous Crying (Crying_S_) and Vowels (‘staccato vowels’). Darker shading on the spectrogram represents higher intensity

The current study thus explored sex identification from naturalistic volitional and spontaneous vocal signals: In a first experiment, participants performed a speaker sex identification task on Spontaneous Laughter (Laughter_S_), Spontaneous Crying (Crying_S_), and cowels (‘staccato vowels’; see Fig. 1 for example waveforms and spectrograms). Given previous findings of impaired person perception from spontaneous vocalizations (Lavan et al. 2016b, 2018a), we hypothesized that the perception of speaker sex would be impaired for spontaneous (emotional) vocalizations, with listeners’ performance for Laughter_S_ and Crying_S_ being significantly less accurate than for Vowels, while performance for Laughter_S_ and Crying_S_ should be similar. We furthermore predicted that these effects should be reflected in reaction times: speaker sex perception from spontaneous vocalizations should be associated with increased task difficulty, which would lead to longer reaction times. Based on previous studies highlighting the importance of F0 on speaker sex perception, we also expected that changes in performance could be linked to variation in F0 between the different types of vocalization.

## Experiment 1

### Method

#### Participants

44 participants (24 female; *M*_*Age*_ = 20.9 years; *SD* = 1.2 years) were recruited at the Department of Psychology at Royal Holloway, University of London and received course credit for their participation. All participants had normal or corrected-to-normal vision and did not report any hearing difficulties. Ethical approval was obtained from the Departmental Ethics Committee at the Department of Psychology at Royal Holloway University of London. None of the participants was familiar with the speakers used.

#### Materials

Laughter_S_, Crying_S_ and Vowels were recorded from 5 speakers (3 male, 2 female, age range: 23–46 years) in a soundproof, anechoic chamber at University College London. Recordings were obtained using a Bruel and Kjaer 2231 Sound Level Meter, recorded onto a digital audio tape recorder (Sony 60ES; Sony UK Limited, Weybridge, UK) and fed to the S/PDIF digital input of a PC sound card (M-Audio Delta 66; M-Audio, Iver Heath, UK) with a sampling rate of 22,050 Hz. The speakers were seated at a distance of 30 cm at an angle of 15° to the microphone. Laughter_S_ was elicited from speakers while watching or listening to amusing sound or video clips (see McGettigan et al. (2015) for a detailed description of the recording procedure). For Crying_S_, speakers recalled upsetting events and/or initially posed crying to encourage a transition into spontaneous crying associated with genuine felt sadness. Crucially, based on informal questions, each speaker reported genuine feelings of amusement and sadness during and after these recording sessions.

In a pilot study, a group of listeners *(N* = 13) provided ratings of arousal (“*How aroused is the person producing the vocalization?*”, with 1 denoting “*the person is feeling very sleepy and drowsy*” and 7 denoting “*the person is feeling very alert and energetic*”), valence (“*How positive or negative is the person producing this vocalization feeling?*”, with 1 denoting “*very negative*” and 7 denoting “*very positive*”), control over the vocalizations (“*How much control did the person have over the production of the vocalization?*”, with 1 denoting “*none at all*” and 7 denoting “*full control*”) and authenticity (“*How authentic is the vocalization?*”, with 1 denoting “*not authentic at all*” and 7 denoting “*very authentic*”). Note that volitional laughter and crying were included in this pilot study as well. These pilot ratings established that participants reliably rate spontaneous laughter and crying as higher in arousal and authenticity, lower in control over the production of the vocalization, and more extreme in valence (more positive for laughter and more negative for crying, respectively) than their volitional counterparts. The speakers also produced series of short vowels (‘staccato vowels’; /a/, /i/, /e/, /u/, /o/, average vowel duration within a series = .35 s) with a relatively stable pitch (*F0 Mean*, males; *mean* = 140.12 Hz, *SD* = 28.5 Hz; females; *mean* = 250.61 Hz; *SD* = 33.08) to preserve a percept of neutral affective valence. This type of volitional, non-emotional stimulus was chosen as its acoustic structure broadly resembles laughter and crying, given all three vocalizations are based on series of vocalic bursts (see Fig. 1). Individual vocalization exemplars were extracted from the recordings and normalized for RMS amplitude using PRAAT (Boersma and Weenink 2010).

Based on the ratings collected for a larger set of vocalizations in the pilot study, 25 stimuli per vocalization (5 per speaker) were selected, choosing series of vowels that were neutral in valence (*M*_*Valence*_= 3.92; *SD* = .16) and low in arousal (*M*_*Arousal*_= 2.68; *SD* = .29) and spontaneous laughter and crying exemplars that were high in arousal (*M*_*CryingS*_= 3.79, *SD* = .42; *M*_*LaughterS*_= 4.78, *SD* = .76; *t*[48] = 5.69, *p *< .001, Cohen’s *d* = 1.643), and authenticity (*M*_*CryingS*_ = 3.58, *SD* = .81; *M*_*LaughterS*_ = 4.79, *SD *= .90; *t*[48] = 5.02, *p* < .001, Cohen’s *d* = 1.449)—note that the stimulus set did not allow for a match of arousal or authenticity for Laughter_S_ and Crying_S_. All three vocalization sets were matched for duration (*M*_*Vowels*_ = 2.55 s, *SD* = .28; *M*_*CryingS*_ = 2.61 s, *SD* = .30; *M*_*LaughterS*_= 2.47 secs, *SD* = .36; *F*(2,48) = 1.31, *p* = .280, *η*_*p*_^2^ = .052).

A detailed analysis of the acoustic features of the stimuli can be found in the “Appendix” section. Note that all instances of laughter and crying used in the experiments reported here included voiced portions to allow us to measure F0. Such voiced vocalizations represent only a subset of laughs and cries and many unvoiced variants of the vocalization have been described elsewhere (for laughter see Bachorowski and Owren 1999).

#### Procedure

Participants were seated in front of a computer screen. Auditory stimuli were presented at a comfortable volume via headphones (Sennheisser HD 201), using MATLAB (Mathworks, Inc., Natick, MA) with the Psychophysics Toolbox extension (http://psychtoolbox.org/). Participants were presented with 75 stimuli in total (25 per vocalization; Vowels, Laughter_*S*_, and Crying_*S*_) in fully randomized order. During the presentation of the sounds, a fixation cross was shown on the screen, which was then replaced by a prompt asking participants to indicate whether the speaker was male or female (two-way forced choice) via a keyboard press. All trials were timed, giving participants 2.5 s to make a response before automatically moving on to the next trial. Participants were asked to respond as quickly as possible based on their first impression. Reaction times were recorded from the offset of the sound. The data collected was checked for item-effects: item-wise accuracy scores all fell within 3 standard deviations of the vocalization-specific means, thus no items were excluded from further analyses.

## Results

### Speaker Sex Perception from Spontaneous Laughter, Spontaneous Crying and Vowel Sounds

**Fig. 2:**
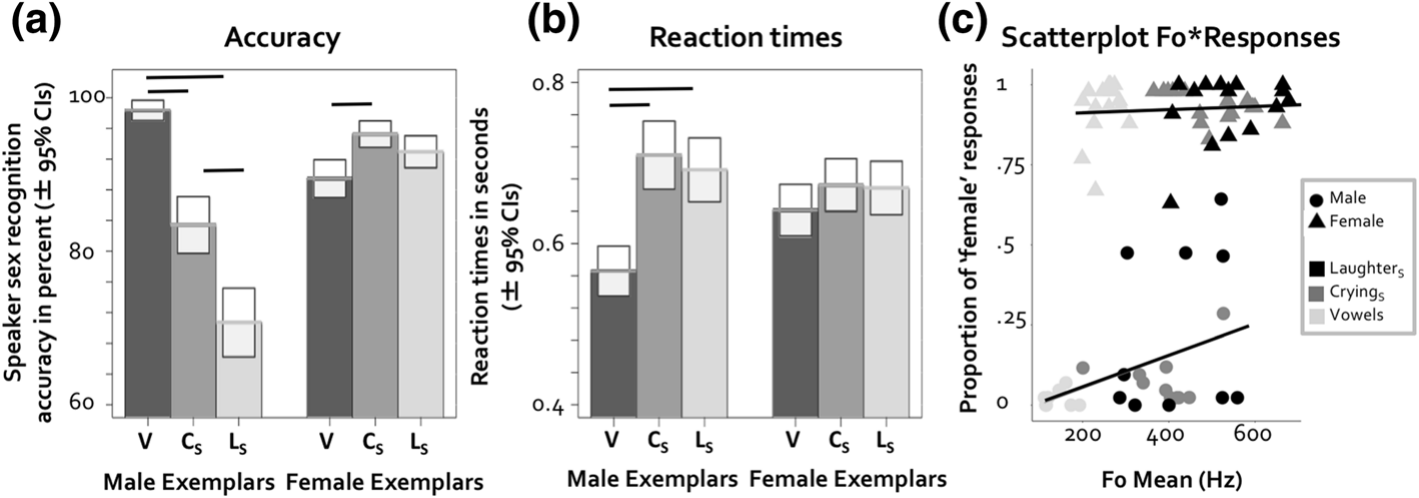
**a** Average accuracy scores per vocalization for the sex identification task of Experiment 1 (*N* = 44) V = Vowels, C_S_ = Crying_S_, L_S_ = Laughter_S_. **b** Average reaction times per vocalization for the sex identification task of Experiment 1. **c** Scatterplot of proportion of ‘female’ responses per item and F0 mean per item. Significant results (*p* < .017) in panels (**a**) and (**b**) are highlighted

To explore whether sex recognition differed for the three vocalizations, we ran a generalized linear mixed effects analysis using *lme4* (Bates et al. 2014) in the *R* environment (R Core Team 2013). We defined one model that predicted binary accuracy codes (correct versus incorrect) based on vocalization type, speaker sex, speaker, and participant. Speaker and participant were entered as random effects. As vocalizations associated with higher F0 (here: Laughter_S_ and Crying_S_ versus Vowels) may lead to differential effects on accuracy for male versus female vocalizations (e.g., Bishop and Keating, 2012; Honorof and Whalen 2010), we included an interaction between speaker sex and vocalization type as a fixed effect in the model. Some of the variance was explained by both the speaker effect (*variance *= .984, *SD *= .992) and participant effect (*variance* = .033, *SD* = .183). Statistical significance was established by likelihood ratio tests contrasting the full model (including the fixed effects, vocalization type, plus the random effects) with a model that did not include the interaction term (Winter 2013). The interaction between speaker sex and vocalization was highly significant (*χ*^2^[2] = 132.32, *p* < .001). For male vocalizations, post hoc planned contrasts between vocalizations by speaker sex (alpha corrected for 6 comparisons) were computed using the R package *lsmeans* (Lenth 2016). These showed that accuracy was significantly higher for Vowels compared to Laughter_S_ and Crying_S_ (*Zs* > 6.53, *p* < .001, estimates > 2.67), which is in line with our predictions that performance should be worse for spontaneous vocalizations. Against predictions, accuracy was furthermore lower for Laughter_S_ compared to Crying_S_ (*Z* = 4.69, *p* < .001, estimate = .90, see Fig. 2a). For female vocalizations, a different pattern of results emerged, where performance for Vowels was significantly worse compared to Crying_S_ (*Z* = 3.86, *p* = .001, estimate = .88), while the remaining two comparisons did not reach significance *Zs* < 2.22, *p* > .026, estimate < .08).

We ran a further linear mixed effects analysis on reaction times that mirrored the accuracy analysis (see Fig. 2b). Little variance was explained by the speaker effect (*variance *= .002, *SD *= .044) or participant effect (*variance* = .041, *SD* = .204). The models showed that the interaction of vocalization type and speaker sex was significant (*χ*^2^[2] = 15.792, *p* < .001). For the planned post hoc contrasts, degrees of freedom were calculated using the Satterthwaite approximation using the *lsmeans* (Lenth 2016). In line with our predictions, these post hoc contrasts showed that reaction times were significantly faster for Vowels compared to Laughter_S_ and Crying_S_ when the vocalizations were produced by males (Laughter_S_—Vowels: *t*[3109.97] = 6.09, *p* < .001, estimate = .12; Crying_S_—Vowels: *t*[3109.87] = 5.089, *p* < .001, estimate = .15). Reaction times for Laughter_S_ and Crying_S_ were comparable (*t*[3109.97] = 1.01, *p* = .311, estimate = .02). The three planned contrasts for vocalizations produced by females were not significant (*t*s < 1.6, *ps* > .109, estimates < .03). For full model specifications and outputs, please see the “Appendix” section.

Taken together, these analyses partially support the prediction that speaker sex perception is more difficult for spontaneous vocalizations, in this case Laughter_S_ and Crying_S_, compared to a volitional vocalization (here, series of vowels). The predicted pattern was, however, only apparent for vocalizations produced by males. Additionally, and against predictions, we also found differences between Laughter_S_ and Crying_S_. These speaker sex-specific can be explained by systematic differences in F0 between male and female voices: laughter and crying both show increased F0 for male and female speakers from to vowel stimuli (see “Appendix” section). Increased F0 in male vocalizations has been shown to lead listeners to perceive such signals as coming from a female (Bishop and Keating 2012; Honorof and Whalen, 2010); however, for female vocalizations, a higher F0 will not lead to such changes in speaker sex perception. Both of these trends are reflected in the results reported above.

### Linking Speaker Sex Judgement Responses to F0

To further explore whether F0 was a salient cue for sex perception from the three vocalizations, we attempted to link the mean F0 of each stimulus to participants’ responses in the speaker sex perception task, using a generalized linear mixed model. Initially, we defined a model with trial-wise raw responses (male vs. female) as the dependent variable, and F0 mean per item and speaker sex as fixed factors. F0 mean was scaled and centered. Based on acoustic consequences of the sexual dimorphism of the vocal tract in humans and the results from the accuracy analyses, we hypothesized that higher Fo in females should increase ‘female’ responses, while higher Fo in males should decrease ‘male’ responses (e.g., Bishop and Keating 2012; Honorof and Whalen 2010). We therefore also modeled an interaction between speaker sex and F0 mean, mirroring the analyses of accuracy and reactions times described above. Vocalization type, speaker, and participant were entered as random factors. Some variance was explained by both the speaker effect (*variance *= 1.248, *SD *= 1.117), and vocalization type (*variance* < .001, *SD* < .001) and participant effect (*variance* = .318, *SD* = .564). Significance of the fixed effects was determined via model comparisons, where the full model was compared to a reduced model (full model minus the interaction). As predicted, there was an interaction between F0 mean and speaker sex for participants’ responses (*χ*^2^[1] = 39.24, *p* < .001). Thus, the increase in “female” responses with increasing F0 was more pronounced for vocalizations produced by males than was the case for vocalizations produced by females (see Fig. 2c). Further models for all male trials as well as all female trials established that the trends for male and female vocalizations were significant (*χ*^2^*s*[1] > 10.4, *ps* < .002).

## Discussion

In the current experiment, we explored whether two emotional vocalizations produced under reduced volitional control would affect the perception of speaker sex from different vocal signals. There were marked differences in how vocalizations produced by males versus females were affected: Performance was impaired for spontaneous male vocalizations, that is for Laughter_*S*_ and Crying_*S*_ compared to Vowels, following our predictions. For female vocalizations, this pattern was, however, not apparent. For male vocalizations, reaction times were furthermore significantly longer for spontaneous versus volitional vocalizations (here: Vowels), indicating greater task difficulty for sex judgements from spontaneous vocalizations.

Such impaired performance for the perception of speaker characteristics has previously been reported for speaker discrimination tasks: listeners were less successful at correctly discriminating speakers from spontaneous laughter compared to volitional laughter (Lavan et al. 2016b), even when this laughter was matched to volitional laughter in perceived authenticity and arousal (Lavan et al. 2018a). As is the case with cues to speaker identity, cues to speaker sex that are encoded within the same acoustic properties are also affected in spontaneous vocalization production, thus being ‘overwritten’ or perceptually less salient. Previous research has shown that F0 and, to a lesser degree, formant measures are perceptually salient cues for the identification of speaker sex (Bishop and Keating 2012; Gelfer and Bennett 2013; Honorof and Whalen 2010; Lass et al. 1976; Mullenix et al. 1995). For speaker sex, global modulations of F0 for laughter and crying result in less marked differences between male and female laughter. These changes in diagnostic cues could explain the current results: F0 for laughter and crying produced by males is matched or may even exceed F0 values usually associated with female vocalizations in the context of speech sounds (> 350 Hz, see “Appendix” section). Lower pitch F0 in vocal signals is generally associated with male speakers, while higher F0 is associated with vocalizations produced by females. Salient acoustic features, such as F0, are modulated drastically in males for spontaneous vocalizations, approximating (and at times exceeding, see “Appendix” section) F0 values frequently encountered in spoken vocalizations produced by females, thus making sex judgements for spontaneous male vocalizations less reliable. For female vocalizations of increasing F0, no such “category boundary” for speaker sex is crossed, explaining the lack of clear effects for this group of stimuli. This interpretation of the data was further backed up by analyses showing that a higher F0 leads to relatively more identifications of male vocalizations as female, compared to vocalizations produced by females. Our study thus shows that naturalistic modulations of salient acoustics features, such as F0, can disrupt speaker sex perception in spontaneous non-verbal vocalizations.

Despite Laughter_S_ and Crying_S_ being spontaneous vocalizations, performance for Laughter_S_ was significantly lower compared to Crying_S_: this may be an indication of vocalization-specific effects although the underlying mechanisms cannot be further probed in this data set. Alternatively, the effect could also be driven by continous perceived affective properties of the vocalizations: Laughter_S_ was significantly higher in percevied arousal than Crying_S_, which could explain the pattern of results. Notably, there are also close links between arousal and F0 (e.g., Juslin and Laukka 2003; Lavan et al. 2016a).

While performance was impaired for spontaneous vocalizations produced by males, mean accuracy was nonetheless high for most vocalizations (raw accuracy > 70%, being close to 100% for some conditions). In line with previous studies that report above-chance accuracy for judgements of speaker sex despite acoustic manipulations of the signal (Bishop and Keating, 2012; Honorof and Whalen 2010; Lass et al. 1995; Mullenix et al. 1995), this current finding confirms that the perception of speaker sex remains largely robust, despite drastic changes introduced to the signal: if one salient acoustic cue such as F0 is modulated to become relatively less salient and diagnostic, acoustic cues such as formant frequencies may still remain informative to listeners and gain importance during perception (e.g., Gelfer and Bennett 2013; Smith and Patterson 2005).

From the current experiment, it cannot yet be determined whether changes in performance are due to differences in the degree of volitional control over voice production (spontaneous vs. volitional), effects of vocalization type (vowels vs. laughter vs. crying), or effects of perceived arousal. In Experiment 2, we addressed these issues by contrasting volitional and spontaneous laughter, which can be classed as a single type of vocalization but which differ in the degree of affective tone and volitional control. If differences in vocalization type modulate performance, performance for Laughter_S_ and Laughter_V_ should be comparable, while performance for Vowels should differ. However, if reduced volitional control over production modulates performance, performance for Vowels and Laughter_V_ should be equivalent, and higher than for Laughter_S_. If perceived arousal drives the effects, sex recognition accuracy should mirror the pattern of perceptual properties of the sounds (i.e., high arousal should be linked to decreases in performance). In line with the previous experiment, we predicted that these effects should be reflected in reaction times and that the accuracy for speaker sex judgements can be linked to the F0 of the vocalizations.

## Experiment 2

### Participants

43 participants (39 female; *M*_*Age*_: 19.2 years; *S*D: 1.1 years) were recruited at the Department of Psychology at Royal Holloway, University of London and received course credit for their participation. No participant reported any hearing difficulties. Ethical approval was obtained from the Departmental Ethics Committee. None of the participants was familiar with the speakers used.

### Materials

Materials were the same as in Experiment 1, with the exception that Crying_S_ was replaced by Laughter_V_ produced by the same 5 speakers (see Experiment 1). The procedure for the recording and elicitation procedure was as described in McGettigan et al. (2015). In short: For Laughter_V_, the speakers were instructed to produce natural and positive sounding laughter, without inducing a specific affective state. Thus, Laughter_V_ was produced with full volitional control over the voice (and in the absence of amusement), while Laughter_S_ was produced spontaneously and thus under reduced volitional control, in response to viewing and listening to amusing stimuli. Laughter_V_ was recorded in the same session as Laughter_S_, with Laughter_V_ always being recorded first to avoid carry-over effects. Based on the ratings from the pilot study (see “Experiment 1” section), 25 Laughter_V_ stimuli (5 per speaker) were selected.

There were marked differences in perceived authenticity between Laughter_V_ and Laughter_S_ (Laughter_V_*M* = 3.60, *SD* = .47; Laughter_S_*M *= 4.79, *SD* = .90; *t*[48] = 5.88, *p* < .001, Cohen’s *d* = 1.697). Laughter_S_ and Laughter_V_ were rated as reflecting significantly higher speaker arousal than Vowels (Laughter_V_: *t*[48] = 12.79, *p* < .001, Cohen’s *d* = 3.692; Laughter_S_: *t*[48] = 13.15, *p* < .001, Cohen’s *d* = 3.796), but in close correspondence to each other (Laughter_V_*M* = 4.39, *SD* = .56; Laughter_S_*M* = 4.78, *SD *= .76; *t*[48] = 2.09, *p* = .042, Cohen’s *d* = .603). There was no perceived difference in speaker valence between the laughter types (Laughter_V_*M* = 5.28, *SD* = .33] Laughter_S_*M* = 5.23, *SD* = 1.06; t[48] = .21, *p* = .836, Cohen’s *d* = .061). The overall duration of the stimuli was matched (Vowels *M* = 2.55 s, *SD *= .28; Laughter_V_*M* = 2.32 s, *SD* = .37; Laughter_S_*M* = 2.47 s, *SD* = .36; one-way repeated measures ANOVA: *F*[2,48] = 3.13, *p* = .053, *η*_*p*_^2^ = .115). A detailed analysis of the acoustic features of the stimuli used in this experiment can be found in the “Appendix” section.

### Procedure

The experimental set up was identical to the one used in Experiment 1. Participants were presented with all 75 stimuli (25 per vocalization; Vowels, Laughter_S_, Laughter_V_) in a fully randomized order. Participants were not pre-informed about the inclusion of spontaneous and volitional laughter in the tasks. The data was checked for item-effects: item-wise accuracy scores all fell within 3 SDs of the vocalization-specific means, thus no items were excluded from further analyses.

## Results

### Speaker Sex Perception from Volitional and Spontaneous Laughter

**Fig. 3:**
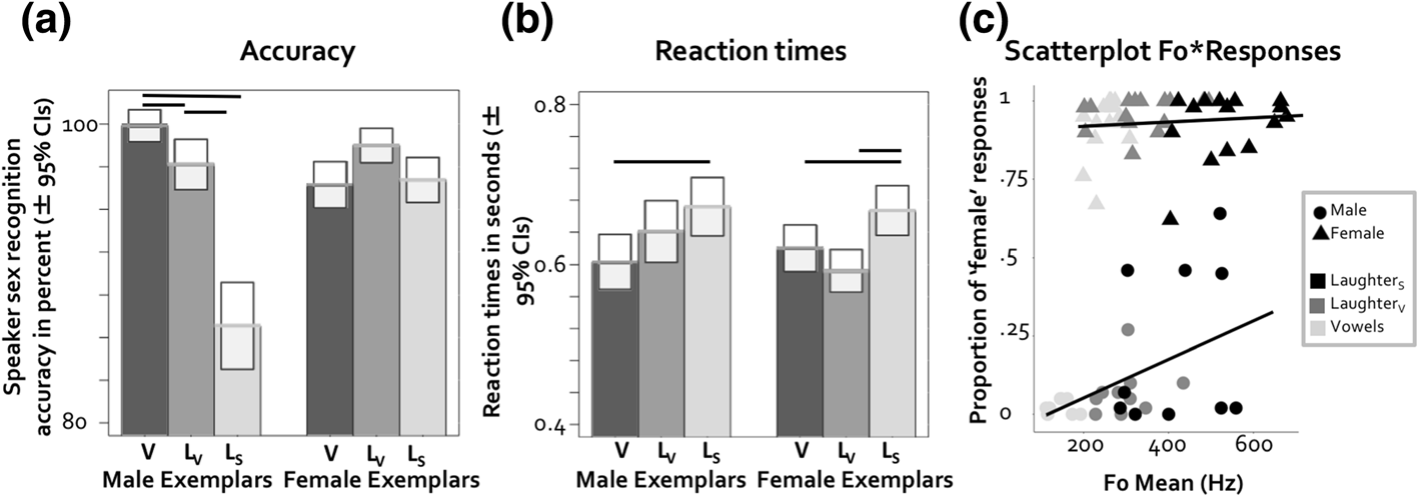
**a** Average accuracy scores per vocalization for the sex identification task of Experiment 2 (*N *= 43). *V* vowels, *L*_*V*_ laughter_v_, *L*_*S*_ laughter_s_. **b** Average reaction times per vocalization for the sex identification task of Experiment 1. **c** Scatterplot of proportion of ‘female’ responses per item and F0 mean per item. Significant results (*p* < .017) in panels (**a**) and (**b**) are highlighted

Data were analyzed with a generalized linear mixed effects analysis of raw accuracy scores. Models were defined in the same way as in Experiment 1: Vocalization type, speaker sex, and an interaction of vocalization type and speaker sex were included as fixed effects, and speaker and participant as random effects. Some of the variance was explained by both the speaker effect (*variance *= 1.177, *SD *= 1.09) and the participant effect (*variance* = .186, *SD* = .43). This analysis confirmed that the interaction between vocalization type and speaker sex were significant (*χ*^2^[2] = 55.73, *p* < .001). In line with our predictions, 6 planned post hoc contrasts showed that accuracy was significantly lower for Laughter_S_ compared to Laughter_V_ and Vowels (both *Z*s > 6.59, both *p*s < .001, estimates > 1.70) for male vocalizations. Against predictions, accuracy for the two volitional vocalizations, Laughter_V_ and Vowels also differed (*Z *= 2.69, *p* = .007, estimate = .108, (see Fig. 3a). Female vocalizations did not follow the predicted pattern and accuracy was highest for Laughter_V_ compared to Vowels and Laughter_S_ (*Zs* > 2.56 2.69, *p* < .011, estimates > .65), while Laughter_S_ and Vowels were similar (*Z* = .288, *p* = .773, estimate = .06).

We ran a further mixed linear effects analysis on reaction times instead of sex recognition accuracy. Here, little variance was explained by both the speaker effect (*variance *= .003, *SD *= .06) and participant effect (*variance* = .025, *SD* = .16). The models showed that vocalization type did have an effect on reaction times: the comparison of the full and the reduced models (minus the effects of interest) was significant (*χ*^2^[2] = 25.239, *p* < .001). Neither speaker sex nor the interaction of vocalization type and speaker sex were significant (*χ*^2^*s *< 5.112, *p* > .077). Six planned post hoc contrasts showed that, for male vocalizations, reaction times were comparable for Vowels and Laughter_V_ (*Z* = 1.66, *p* = .097, estimate = .04) and longer for Laughter_S_ compared to Vowels *(Z* = 3.08, *p* = .002, estimate = .07). While reaction times were numerically longer for Laughter_S_ compared to Laughter_V_, this comparison did not reach significance (*Z* = 1.42, *p* = .154, estimate = .02, see Fig. 3b). For female vocalizations, the planned contrasts confirmed our predictions, with reaction times being comparable for Vowels and Laughter_V_ (*Z* = 1.49, *p* = .137, estimate = .03) but longer for Laughter_S_ and Vowels (*Z* = 2.60, *p* = .102, estimate = .05) and Laughter_S_ and Laughter_V_ (*Z* = 4.06, *p* < .001, estimate = .08).

### Linking Speaker Sex Judgement Responses to F0

In parallel to the analyses conducted for Experiment 1, we initially defined a model with binary participant responses (male vs. female) as the dependent variable and F0 mean per item and speaker sex as fixed factors. We also included an interaction between speaker sex and F0 mean in the model (see “Experiment 1” section). Vocalization type, speaker and participant were entered as random factors. Some variance was explained by both the effect of speaker (*variance *= 1.350, *SD *= 1.162), (*variance* < .001, *SD* < .001) and participant effect (*variance* = .891, *SD* = .944). Significance of the effects was determined via model comparisons, where the full model was compared to a reduced model (full model minus the factors of interest). There was a significant interaction between F0 mean and speaker sex (*χ*^2^[1] = 34.114, *p* < .001). With increasing F0 mean, listeners more frequently chose ‘female’ as a response for vocalizations that were produced by males. This was also true—albeit to a lesser extent—for female vocalizations (see Fig. 3c). Further models for all male trials as well as all female trials established that both of these trends in the data were significant (both *χ*^2^*s*[1] > 20.12, both *p*s < .001).

## Discussion

By contrasting Laughter_V_ and Laughter_S_, Experiment 2 explored whether the effects observed in Experiment 1 reflected processing differences for different types of vocalizations (laughter vs. vowels), whether they could have resulted from differences in production mode (volitional vs. spontaneous) or, finally, whether they reflected differences in perceived arousal. As in Experiment 1, we have seen clear differences in patterns of results for male versus female speakers. For male vocalizations, accuracy was lower for Laughter_S_ than for Vowels and Laughter_V_, while performance was comparable for Vowels and Laughter_V_. Accuracy was also lower for Laughter_V_ compared to Vowels, although this effect was notably smaller compared to the effect of Laughter_S_ versus Laughter_V_. The current results thus indicate that reduced volitional control has an effect on the perception of speaker sex for male vocalizations, echoing findings from speaker discrimination tasks (Lavan et al. 2016b, 2018a): if perceived arousal would have a substantial effect on speaker sex recognition, we should have seen Laughter_V_ behaving more like Laughter_S_ (where overall differences in arousal were comparatively small) and less like Vowels (where arousal differences were more pronounced). Our findings, however, show the opposite for male vocalizations. In line with the results of Experiment 1, a relationship between F0 and speaker sex recognition accuracy was found: listeners were more likely to judge vocalizations as being produced by a female speaker when F0 increased, and this effect was more pronounced for vocalizations produced by males.

## General Discussion and Limitations

In the current set of experiments, we investigated whether the perception of speaker sex from non-verbal vocalizations is affected by natural vocal flexibility, introduced by using different types of vocalizations (laughter, crying, vowels) produced under different levels of volitional control (spontaneous versus volitional emotional vocalizations). We found striking interactions of speaker sex with accuracy: while our predictions were largely confirmed for male vocalizations, this was not the case for female vocalizations. For male vocalizations, our results indicate that accuracy is lower for spontaneous compared to volitional vocalizations, with graded differences being apparent for different types of spontaneous vocalizations (see Experiment 1: better performance for Crying_S_ compared to Laughter_S_). These results are in line with the findings of two recent studies of speaker discrimination using spontaneous and volitional vocalizations (Lavan et al. 2016b, 2018a): In these studies, listeners’ ability to determine whether a pair of vocalizations was produced by the same or two different speakers was significantly worse for spontaneous laughter compared to volitional laughter. While no clear link between acoustic cues and discrimination performance could be found in the previous studies, the current experiments found links between F0 and speaker sex perception accuracy. Acoustic cues that are diagnostic for speaker sex in neutral vocal signals are drastically modulated during the production of emotional vocalizations, rendering these acoustic cues less diagnostic. In this study, vocalizations with high F0—especially those produced by males—were more likely to be perceived as being produced by females; the opposite pattern also held for female vocalizations, albeit more weakly and in the presence of a ceiling effect for higher F0. While F0 is known to be a salient cue for speaker sex judgements from (neutral) volitional speech sounds, its role and importance in determining speaker sex is largely unknown for other types of vocalizations. The current study suggests that F0 also appears to be an important acoustic cue to speaker sex in volitional and spontaneous vocalizations, such as laughter and crying. Due to this perceptual salience, naturalistic modulations of F0 can impair speaker sex perception in emotional vocalizations, especially when F0 values go beyond what can be considered to be the “typical” range for a category (here male vs. female).

There are, however, a number of limitations to the current study that should be noted. First, we only used 5 different speakers, which can be a considered a relatively low number. We would argue that it is unlikely that participants were aware of the small number of speakers given the inclusion of different vocalizations: a study has reported that unfamiliar listeners are unable to discriminate between speakers when making judgements across different kinds of vocalizations (such as laughter and vowels, Lavan et al. 2016b) and tend to assume that more voices than actually included in a stimuli set (Lavan et al. 2018c, 2018b). Second, the acoustic analysis focused solely on linking F0 to sex perception accuracy. While F0 is arguably the most important acoustic cue to speaker identity (Gelfer and Bennett 2013; Poon and Ng 2015; Whiteside 1998), formant measures have frequently also been implicated. The current study did not extract any formant measures. While previous studies have extracted formant measures from nonverbal emotional vocalizations, such as laughter (e.g., Szameitat et al. 2009a, b; Bachorowski et al. 2001), they came to conflicting conclusions. For most vocalizations, especially for spontaneous ones, the authors of those studies report that it was difficult to extract reliable formant measures from a representative portion of the sounds (see Bachorowski et al. 2001, for a discussion). An analysis of such formant measures would thus have lacked adequate precision, and was omitted from the current experiments.

The expression and perception of speaker sex has been discussed extensively in the literature on human voice perception, with particular emphasis on the marked sexual dimorphism as being distinct and exaggerated compared with other species (Titze 1989). Vocal cues, such as F0, have thus been reported to play a role in sexual selection (e.g., Puts et al. 2016): In our study, we investigated the effects of natural variability in vocal behavior on the identification of sex from vocalizations produced by adult male and female speakers and find that perceptual performance is significantly impaired when vocalizations are produced under reduced volitional control. Furthermore, the sexual dimorphism is drastically reduced between males and females for the spontaneous vocalizations used in this study (see “Appendix” section for a full breakdown of acoustic properties of the stimuli). This work thus calls for further discussions of the role of acoustic cues such as fundamental frequency in the signalling of speaker sex (and e.g., reproductive fitness) in the context of vocal flexibility, and how the expression of these signals may be particularly dependent on the modern human’s capacity for controlled vocal behavior (see Lavan et al. 2018a).

Furthermore, this work is of interest to the forensic literature, where studies have shown that earwitness speaker recognition or identification is notoriously unreliable (Clifford 1980). It has also been shown that listeners struggle to match emotional speech to neutral speech across a time delay (Read and Craik 1995; Saslove and Yarmey 1980). Our study adds to and partially extends these findings: a perpetrator may dramatically modulate their F0—through voice disguise (see also Wagner and Köster 1999) or, as is the case in this study, spontaneously so when experiencing intense emotions that may occur at a crime scene. In such a scenario, not only are explicit judgements about the identity of a potential perpetrator unreliable, but more basic judgements such as speaker sex may also at times be affected. Further, the work is relevant to computational speaker recognition or verification: the robustness and reliability of such algorithms is determined by the type of training they received to build up a template for a speaker’s voice. The current study indicates that in the context of vocal flexibility, human listeners can fail to reliability make the relatively basic judgement of speaker sex. Algorithms neglecting the presence of vocal flexibility in training sets or relying on just a single verification phrase may thus become unreliable for (spontaneous) emotionally-inflected vocal signals.

Overall, the current study shows how the flexibility of our voices affects perceptual judgements. A complex picture of speaker sex-specific effects emerged, interacting with our experimental manipulation that contrasted volitional and spontaneous vocalizations. Generalizations about how vocal signals behave at large can be problematic and may overlook nuanced effects that shape and characterise human voice processing.

## Appendix

### Acoustic Features of the Stimuli of Experiment 1

Table 1 shows an overview of the means for the acoustic properties of the stimuli. The following acoustic measures were used:

**Table 1:** Acoustic measures of the stimuli used in Experiment 1

					By Gender			
			All		Male		Female	
Vocalisation	Acoustic measure	Unit	Mean	SD	Mean	SD	Mean	SD
Vowels	Duration	s	2.55	0.28	2.41	0.25	2.64	0.27
	Burst duration (mean)	s	0.36	0.10	0.34	0.12	0.37	0.08
	Unvoiced segments	%	22.24	13.77	25.48	10.89	20.08	15.37
	F0 (mean)	Hz	206.42	63.21	140.12	28.5	250.61	33.08
	F0 (SD)	Hz	78.33	52.95	54.1	44.29	94.48	53.35
	Spectral center of gravity	Hz	688.13	370.35	786.15	551.51	622.79	167.79
	HNR	Hz	17.91	4.87	13.89	3.77	20.59	3.5
	Jitter	dB	1.28	0.54	1.57	0.48	1.08	0.50
	Shimmer	dB	0.58	0.22	0.67	0.23	0.53	0.21
Crying_S_	Duration	s	2.61	0.30	2.70	0.33	2.56	0.27
	Burst Duration (mean)	s	0.19	0.13	0.15	0.05	0.21	0.16
	Unvoiced segments	%	53.86	16.69	52.59	15.65	54.70	17.84
	F0 (mean)	Hz	454.95	102.64	387.88	85.39	499.67	89.54
	F0 (SD)	Hz	108.21	40.08	117.22	35.9	102.21	42.77
	Spectral center of gravity	Hz	687.55	172.33	577.97	146.54	760.6	151.03
	HNR	Hz	11.16	5.05	9.81	2.73	12.06	6.07
	Jitter	dB	2.98	1.35	3.76	1.36	2.46	1.11
	Shimmer	dB	1.12	0.37	1.2	0.17	1.06	0.45
Laughter_S_	Duration	s	2.47	0.35	2.75	0.14	2.28	0.31
	Burst duration (mean)	s	0.11	0.06	0.14	0.08	0.1	0.04
	Unvoiced segments	%	58.82	14.75	57.27	14.32	59.85	15.45
	F0 (mean)	Hz	490.23	115.89	417.65	109.96	538.61	94.77
	F0 (SD)	Hz	113.22	36.78	115.46	34.7	111.73	39.23
	Spectral center of gravity	Hz	1047.98	372.7	838.38	260.52	1187.72	377.08
	HNR	Hz	9.36	2.72	9.61	1.69	9.19	3.28
	Jitter	dB	3.05	1.11	3.13	0.64	3.00	1.36
	Shimmer	dB	1.15	0.25	1.16	0.14	1.14	0.30

1. *Duration* The interval between the first zero-crossing of the onset to the final zero crossing after the offset of the vocalization.
2. *Burst duration* The interval between the first zero-crossing of the onset to the final zero crossing of a vocalic burst.
3. *Percentage of unvoiced segments* Percentage of frames lacking harmonic structure.
4. *F0 mean* Computed using the auto-correlation method in PRAAT. F0 floor was set at 75 Hz and the F0 ceiling at 1000 Hz.
5. *F0 standard deviation* The standard deviation of the F0 mean.
6. *Spectral centre of gravity* Measure for the mean height of the frequencies for each vocalization, which captures the weighting of energy in the sound across the frequency range.
7. *Mean harmonics-to-noise-ratio (HNR)* The mean ratio of quasi periodic to non-period signals across time segments.
8. *Jitter* The average absolute difference between consecutive periods, divided by the average period, i.e., micro-fluctuations in the duration of each period.
9. *Shimmer* The average absolute difference between the amplitudes of consecutive periods, divided by the average amplitude.

Independent samples *t* tests were performed to assess acoustic differences between vocalizations (alpha was correct for 9 comparisons). These tests showed that while laughter_S_ and crying_S_ were acoustically similar for all acoustic measures (all *p*s > .016) with the exception of spectral center of gravity (*p* < .001), crying_S_ differed from vowels all acoustic measures (*p* < .001) with the exception of total duration (*p* = .425), F0 *SD* (*p* = .029) and spectral center of gravity (*p* = .994). The acoustic properties of Laughter_S_ were significantly different from vowels for all measures (*p* < .002), except F0 *SD* (*p *= .009) total duration (*p* = .364). Despite constituting two different vocalizations, laughter_S_ and crying_S_ can be thus considered acoustically more similar to each other, while vowels were acoustically very dissimilar to both laughter_S_ and crying_S_. For a detailed breakdown of the acoustic properties of the stimuli by speaker and by gender, see Table 1.

### Acoustic Features of the Stimuli Used in Experiment 2

Table 2 shows an overview of the means for the acoustic properties of the stimuli. Independent samples t-tests were performed to assess acoustic differences between vocalizations (alpha was correct for 9 comparisons). These tests showed that laughter_S_ and vowels were acoustically distinct for all measures (*p*s < .002) except and F0 *SD* (*p* = .019) and total duration (*p *= .496). Laughter_V_ was also distinct from vowels for all acoustic measures (*p*s < .002) with the exception of spectral center of gravity (*p* = .035) and total duration (*p *= .007). Laughter_S_ and laughter_V_ were similar to each other for a number of acoustic measures (total duration, spectral center of gravity, percentage of unvoiced segments, F0 *SD* and bust duration; all *p*s > .05) and differed significantly from each other for F0 mean, HNR, shimmer, jitter, all *p*s < .002). Thus, while laughter_S_ and laughter_V_ clearly differ from vowels in their acoustics properties, the two types of laughter seem to be acoustically similar for some acoustic features.

**Table 2:** Acoustic measures of the stimuli used in experiment 1

					By gender			
			All		Male		Female	
Vocalisation	Acoustic measure	Unit	Mean	SD	Mean	SD	Mean	SD
Vowels	Duration	s	2.55	0.28	2.41	0.25	2.64	0.27
	Burst duration (mean)	s	0.36	0.1	0.34	0.12	0.37	0.08
	Unvoiced segments	%	22.24	13.77	25.48	10.89	20.08	15.37
	F0 (mean)	Hz	206.42	63.21	140.12	28.50	250.61	33.08
	F0 (SD)	Hz	78.33	52.95	54.1	44.29	94.48	53.35
	Spectral center of gravity	Hz	688.13	370.35	786.15	551.51	622.79	167.79
	HNR	Hz	17.91	4.87	13.89	3.77	20.59	3.50
	Jitter	dB	1.28	0.54	1.57	0.48	1.08	0.50
	Shimmer	dB	0.58	0.22	0.67	0.23	0.53	0.21
Laughter_V_	Duration	s	2.34	0.38	2.15	0.24	2.46	0.41
	Burst duration (mean)	s	0.09	0.02	0.09	0.02	0.09	0.03
	Unvoiced segments	%	58.72	10.34	55.56	11.82	60.76	9.14
	F0 (mean)	Hz	322.74	83.55	299.37	65.09	337.75	92.66
	F0 (SD)	Hz	127.85	60.46	110.38	37.61	139.09	70.48
	Spectral center of gravity	Hz	871.81	278.18	843.1	270.17	890.26	291.72
	HNR	Hz	5.93	1.79	6.41	1.57	5.63	1.92
	Jitter	dB	3.91	0.53	3.62	0.49	4.09	0.47
	Shimmer	dB	1.34	0.26	1.24	0.15	1.40	0.30
Laughter_S_	Duration	s	2.47	0.35	2.75	0.14	2.28	0.31
	Burst duration (mean)	s	0.11	0.06	0.14	0.08	0.10	0.04
	Unvoiced segments	%	58.82	14.75	57.27	14.32	59.85	15.45
	F0 (mean)	Hz	490.23	115.89	417.65	109.96	538.61	94.77
	F0 (SD)	Hz	113.22	36.78	115.46	34.7	111.73	39.23
	Spectral center of gravity	Hz	1047.98	372.70	838.38	260.52	1187.72	377.08
	HNR	Hz	9.36	2.72	9.61	1.69	9.19	3.28
	Jitter	dB	3.05	1.11	3.13	0.64	3.00	1.36
	Shimmer	dB	1.15	0.25	1.16	0.14	1.14	0.30

### Model Outputs

The following section presents the model outputs for the full models, followed by the log likelihood tests described in the main paper.

See Tables 3, 4, 5, 6, 7, 8.

**Table 3:** Experiment 1: accuracy models

Random effects			Fixed effects			
Factor	Variance	SD	Factor	Estimate	SE	*z*
Participant	0.03	0.18				
Speaker	0.98	0.99				
			(Intercept)	4.91	0.81	6.07
			Vocalization type 2	− 2.67	0.41	− 6.53
			Vocalization type 3	− 3.57	0.41	− 8.83
			Speaker Sex 1	− 2.68	1.00	− 2.68
			Vocalization type 2 : speaker sex 1	3.55	0.47	7.59
			Vocalization type 3: speaker sex 1	4.02	0.45	8.88

	*Df*	AIC	BIC	logLik	Deviance	*χ* ^2^	*Df*	*p*
Reduced model: interaction	6	1873.40	1909.80	− 930.72	1861.40			
Full model	8	1745.20	1793.70	− 864.61	1729.20	132.23	2	< .001

Full model: Accuracy ~ Vocalization type + Speaker sex + Vocalization type: speaker sex + (1 | Speaker) + (1 | Participant)

Reduced model (interaction): Accuracy ~ Vocalization type + Speaker sex + (1 | Speaker) + (1 | Participant)

Vocalization type: 1—vowels, 2—crying_s_, 3—laughter_s_

Speaker sex: 0—male, 1—female

**Table 4:** Experiment 1: RT models

Random effects			Fixed effects				
Factor	Variance	SD	Factor	Estimate	SE	*df*	*t*
Participant	.04	.20					
Speaker	<.001	.04					
			(Intercept)	.57	.05	17.93	12.07
			Vocalization type 2	.15	.02	3109.97	6.09
			Vocalization type 3	.12	.02	3109.87	5.09
			Speaker sex 1	.08	.05	6.34	1.68
			Vocalization type 2: speaker sex 1	− .12	.03	3109.94	− 3.71
			Vocalization type 3: speaker sex 1	− .10	.03	3109.92	− 3.09

	*Df*	AIC	BIC	logLik	Deviance	*χ* ^2^	*Df*	*p*
Reduced model: interaction	7	2101.50	2143.80	− 1043.80	2087.50			
Full model	9	2100.40	2154.80	− 1041.20	2082.40	5.11	2	.08
Reduced model: speaker sex	6	2099.60	2135.80	− 1043.80	2087.60			
Full model	9	2100.40	2154.80	− 1041.20	2082.40	5.16	3	.16
Reduced model: vocalization type	5	2118.60	2148.80	− 1054.30	2108.60			
Full model	9	2100.40	2154.80	− 1041.20	2082.40	26.24	4	< .001

Full model: RT ~ Vocalization type + Speaker sex + Vocalization type: Speaker sex + (1 | Speaker) + (1 | Participant)

Reduced model (interaction): RT ~ Vocalization type + Speaker sex + (1 | Speaker) + (1 | Participant)

Reduced model (speaker sex): RT ~ Vocalization type + (1 | Speaker) + (1 | Participant)

Reduced model (vocalization type): RT ~ Speaker sex + (1 | Speaker) + (1 | Participant)

Vocalization type: 1—vowels, 2—crying_s_, 3—laughter_s_

Speaker sex: 0—male, 1—female

**Table 5:** Experiment 1: F0 models

Random effects			Fixed effects			
Factor	Variance	SD	Factor	Estimate	SE	z
Participant	.32	.56				
Speaker	1.25	1.12				
Vocalization type	< .001	<.001				
			(Intercept)	− 2.17	.81	− 2.70
			Speaker sex 1	4.84	1.03	4.69
			F0	1.37	.12	11.71
			Speaker 1: F0	− .94	.15	− 6.20

	*Df*	AIC	BIC	logLik	Deviance	*χ* ^2^	*Df*	*p*
Reduced model: interaction	6	1737.40	1773.80	− 862.72	1725.40			
Full model	7	1700.20	1742.60	− 843.10	1686.20	39.24	1	< .001

Full model: Response ‘Female’ ~ Speaker sex + F0 + F0: Speaker sex + (1 | Vocalizations type) + (1 | Speaker) + (1 | Participant)

Reduced model (interaction): Response ‘Female’ ~ Speaker sex + F0 + (1 | Vocalizations type) + (1 | Speaker) + (1 | Participant)

Speaker sex: 0—male, 1—female

**Table 6:** Experiment 2: accuracy models

Random effects			Fixed effects			
Factor	Variance	SD	Factor	Estimate	SE	*z*
Participant	.19	.43				
Speaker	1.18	1.08				
			(Intercept)	4.78	0.86	5.57
			Vocalization type 2	− 1.08	.40	− 2.70
			Vocalization type 3	− 2.78	.37	− 7.58
			Speaker sex 1	− 2.08	1.07	− 1.94
			Vocalization type 2: speaker sex 1	1.80	0.47	3.80
			Vocalization type 3: speaker sex 1	2.84	.43	6.68

	*Df*	AIC	BIC	logLik	Deviance	*χ* ^2^	*Df*	*p*
Reduced model: interaction	6	1471.30	1507.60	− 729.68	1459.30			
Full model	8	1419.60	1468.00	− 701.81	1403.60	55.73	2	< .001

Full model: Accuracy ~ Vocalization type + Speaker sex + Vocalization Type : Speaker sex + (1 | Speaker) + (1 | Participant)

Reduced model (interaction): Accuracy ~ Vocalization Type + Speaker sex + (1 | Speaker) + (1 | Participant)

Vocalization type: 1—vowels, 2—laughter_v_, 3—laughter_s_

Speaker sex: 0—male, 1—female

**Table 7:** Experiment 2: RT models

Random effects		Fixed effects					
Factor	Variance	SD	Factor	Estimate	SE	*df*	*t*
Participant	0.03	0.16					
Speaker	< .001	0.06					
			(Intercept)	0.60	0.05	9.83	12.06
			Vocalization type 2	0.04	0.02	3064.96	1.66
			Vocalization type 3	0.07	0.02	3065.03	3.08
			Speaker sex 1	0.02	0.06	5.79	0.31
			Vocalization type 2: speaker sex 1	− 0.07	0.03	3064.96	− 2.23
			Vocalization type 3: speaker sex 1	− 0.02	0.03	3065.01	− 0.77

	*Df*	AIC	BIC	logLik	deviance	*χ* ^2^	*Df*	*p*
Reduced model: interaction	7	2582.20	2624.60	− 1284.10	2568.20			
Full model	9	2570.40	2624.90	− 1276.20	2552.40	15.79	2	< .001

Full model: Accuracy ~ Vocalization type + Speaker sex + Vocalization type: speaker sex + (1 | Speaker) + (1 | Participant)

Reduced model (interaction): Accuracy ~ Vocalization type + Speaker sex + (1 | Speaker) + (1 | Participant)

Vocalization type: 1—vowels, 2—laughter_v_, 3—laughter_s_

Speaker sex: 0—male, 1—female

**Table 8:** Experiment 2: F0 models

Random effects			Fixed effects			
Factor	Variance	SD	Factor	Estimate	SE	z
Participant	0.89	0.94				
Speaker	1.35	1.16				
Vocalization type	< .001	< .001				
			(Intercept)	− 3.31	0.86	− 3.86
			Speaker sex 1	6.48	1.09	5.94
			F0	1.23	0.13	9.52
			Speaker 1: F0	− 1.00	0.17	− 5.96

	*Df*	AIC	BIC	logLik	Deviance	*χ* ^2^	*Df*	*p*
Reduced model: interaction	6	1386.3	1422.6	− 687.15	1374.3			
Full model	7	1354.2	1396.5	− 670.1	1340.2	34.114	1	< .001

Full model: Response ‘Female’ ~ Speaker sex + F0 + F0: Speaker sex + (1 | Vocalizations type) + (1 | Speaker) + (1 | Participant)

Reduced model (interaction): Response ‘Female’ ~ Speaker sex + F0 + (1 | Vocalizations type) + (1 | Speaker) + (1 | Participant)

Speaker sex: 0—male, 1—female

## Footnotes

1 Sine-wave speech is manipulated speech that retains only the formants (spectral prominences linked to articulatory movements) from the original speech signal to create a high-pitched, chirping sound can nonetheless be intelligible.

2 Noise-vocoded speech is another manipulated speech signal intelligible speech, which retains the amplitude-modulated temporal information for a limited number of frequency bands resulting something that is perceptually akin to a harsh whisper (Shannon et al. 1995).
